# Supplementary material for: A unified framework for species spatial patterns: Linking the occupancy area curve, Taylor's Law, the neighborhood density function and two‐plot species turnover
Source: Ecol Lett. 2021 Aug 4;24(10):2043–53. doi: 10.1111/ele.13788 (PMC8518128; doi:10.1111/ele.13788)
Supplement: Supplementary file 4 — Supplementary Material [file ELE-24-2043-s004.zip › calcs/6-figures/spp-preds/psycma_preds.pdf]

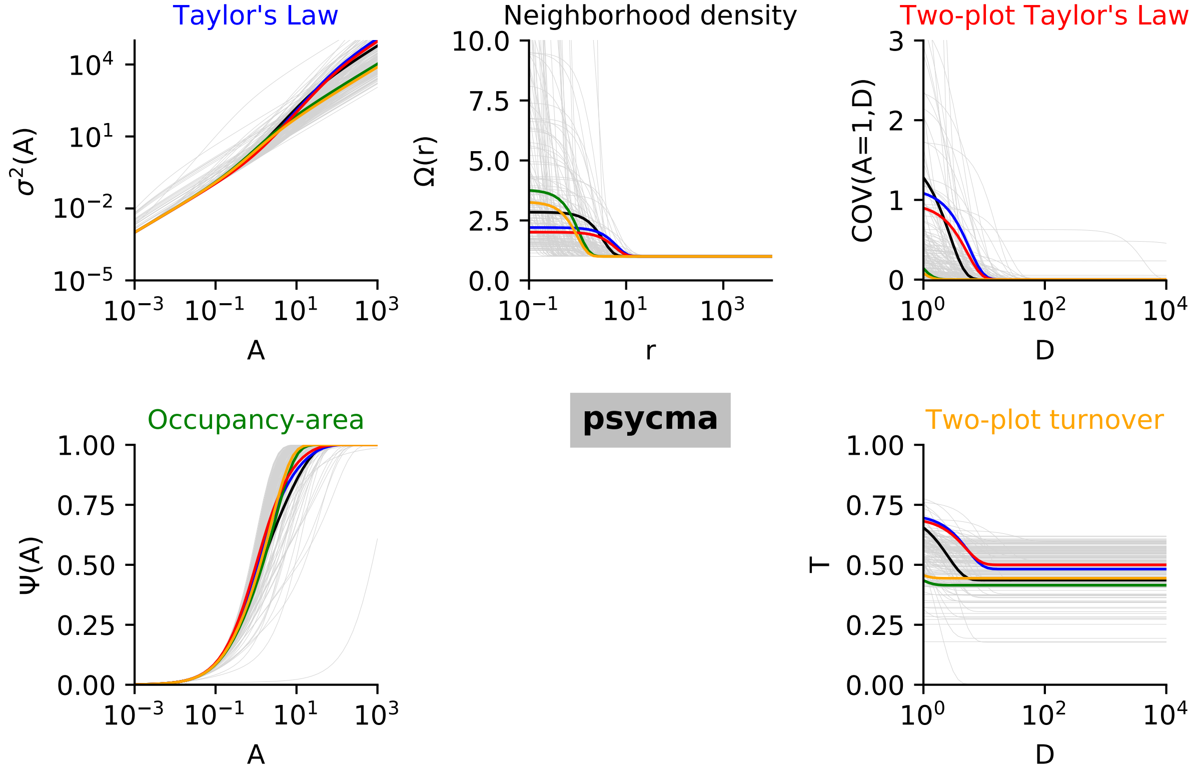

Taylor's Law
σ^2(A)
10^4
10^1
10^{-2}
10^{-5}
10^{-3} 10^{-1} 10^1 10^3
A
Neighborhood density
Ω(r)
10.0
7.5
5.0
2.5
0.0
10^{-1} 10^1 10^3
r
Two-plot Taylor's Law
COV(A=1,D)
3
2
1
0
10^0 10^2 10^4
D
Occupancy-area
Ψ(A)
1.00
0.75
0.50
0.25
0.00
10^{-3} 10^{-1} 10^1 10^3
A
psycma
Two-plot turnover
T
1.00
0.75
0.50
0.25
0.00
10^0 10^2 10^4
D
